# Induction of Apoptosis and Regulation of MicroRNA Expression by (2*E*,6*E*)-2,6-*bis*-(4-hydroxy-3-methoxybenzylidene)-cyclohexanone (BHMC) Treatment on MCF-7 Breast Cancer Cells

**DOI:** 10.3390/molecules26051277

**Published:** 2021-02-26

**Authors:** Swee Keong Yeap, Norlaily Mohd Ali, Muhammad Nadeem Akhtar, Nursyamirah Abd Razak, Zhi Xiong Chong, Wan Yong Ho, Lily Boo, Seema Zareen, Tonni Agustiono Kurniawan, Ram Avtar, Stephanie Y. L. Ng, Alan Han Kiat Ong, Noorjahan Banu Alitheen

**Affiliations:** 1 China-ASEAN College of Marine Sciences, Xiamen University Malaysia Campus, Jalan Sunsuria, Bandar Sunsuria, Sepang 43900, Selangor, Malaysia; skyeap@xmu.edu.my (S.K.Y.); ngyls@xmu.edu.my (S.Y.L.N.); 2 Department of Cell and Molecular Biology, Faculty of Biotechnology and Biomolecular Sciences, University Putra Malaysia, Serdang 43400, Selangor, Malaysia; norlailyma@gmail.com (N.M.A.); mieyra611@gmail.com.my (N.A.R.); 3 Faculty of Medicine and Health Sciences, Universiti Tunku Abdul Rahman, Sungai Long Campus, Jalan Sungai Long, Bandar Sungai Long, Cheras, Kajang 43000, Selangor, Malaysia; boolily83@gmail.com (L.B.); onghk@utar.edu.my (A.H.K.O.); 4 Faculty of Industrial Sciences & Technology, Universiti Malaysia Pahang, Lebuhraya Tun Razak 26300, Kuantan Pahang, Malaysia; nadeemupm@gmail.com (M.N.A.); seema.zareen@gmail.com (S.Z.); 5 School of Biomedical Sciences, The University of Nottingham Malaysia Campus, Jalan Broga, Semenyih 43500, Selangor, Malaysia; khyy5czx@nottingham.edu.my (Z.X.C.); wanyong.ho@nottingham.edu.my (W.Y.H.); 6 Key Laboratory of the Coastal and Wetland Ecosystems (Xiamen University), Ministry of Education, College of the Environment and Ecology, Xiamen University, Xiamen 361102, China; tonni@xmu.edu.cn; 7 Faculty of Environmental Earth Sciences, Hokkaido University, Sapporo 060-0810, Japan; ram@ees.hokudai.ac.jp; 8 UPM-MAKNA Cancer Research Laboratory, Institute of Bioscience, Universiti Putra Malaysia, Serdang 43400, Selangor, Malaysia

**Keywords:** breast cancer, 2,6-*bis*-(4-hydroxy-3-methoxybenzylidene)-cyclohexanone (BHMC), MCF-7, apoptosis, miRNA

## Abstract

(2*E*,6*E*)-2,6-*bis*-(4-hydroxy-3-methoxybenzylidene)-cyclohexanone (BHMC) is a synthetic curcumin analogue, which has been reported to possess anti-tumor, anti-metastatic, and anti-invasion properties on estrogen receptor (ER) negative breast cancer cells in vitro and in vivo. However, the cytotoxic effects of BHMC on ER positive breast cancer cells were not widely reported. This study was aimed to investigate the cytotoxic potential of BHMC on MCF-7 cells using cell viability, cell cycle, and apoptotic assays. Besides, microarray and quantitative polymerase chain reaction (qPCR) were performed to identify the list of miRNAs and genes, which could be dysregulated following BHMC treatment. The current study discovered that BHMC exhibits selective cytotoxic effects on ER positive MCF-7 cells as compared to ER negative MDA-MB-231 cells and normal breast cells, MCF-10A. BHMC was shown to promote G2/M cell cycle arrest and apoptosis in MCF-7 cells. Microarray and qPCR analysis demonstrated that BHMC treatment would upregulate several miRNAs like miR-3195 and miR-30a-3p and downregulate miRNAs such as miR-6813-5p and miR-6132 in MCF-7 cells. Besides, BHMC administration was also found to downregulate few tumor-promoting genes like VEGF and SNAIL in MCF-7. In conclusion, BHMC induced apoptosis in the MCF-7 cells by altering the expressions of apoptotic-regulating miRNAs and associated genes.

## 1. Introduction

Breast cancer remains as a prominent oncological disease worldwide by making up 14% of the total cancer deaths in 2008 [1] and it is the most prevalent cancer that affects women worldwide [2]. Breast cancer can be classified into different sub-types based on their molecular features [3] and one of these features is the presence of estrogen receptor (ER) [4]. With the advancement in the breast cancer diagnostic and management technology, incidences of breast cancer were found to show slower increase since year 2000 [1]. Compared to the incidence of ER negative breast cancer, which was estimated to be decreasing, the incidence of ER positive cancer was estimated to show an increasing trend from year 2009 to 2016 [4]. Despite the success of endocrine treatment, not all ER-positive breast cancer patients are responsive to this first line treatment and some of them might eventually face relapse or recurrence [5]. Thus, it is crucial to identify novel potential therapeutic agent to treat ER positive breast cancer. Besides, more study should be conducted to unravel the detailed biological processes and signaling pathways that lead to the development of ER positive breast cancer as these could help improve the endocrine therapy efficacy in treating ER positive breast cancer.

Indeed, natural products are known as powerful resources for drug discovery and development [6]. It has contributed for approximately 36% of the US Food and Drug Administration (FDA)-approved compounds in between 1998 and 2008 [7]. Among these natural products, curcumin is a natural dietary pigment present in the root of turmeric *Curcuma Longa* [8]. It has been well documented to possess anti-tumor and anti-metastatic properties against breast cancer [9]. A previous study has reported that curcumin is more selective on ER negative breast cancer cells due to the differential regulation of SKP2-CIp/Kips signaling pathway [10]. Rapid development in the chemical synthesis technology advances the potential to discover novel, synthetic anti-cancer compounds as it overcomes the supply problems in getting similar compounds from natural products, and it also helps to discover anti-cancer compounds with improved cytotoxicity and selectivity via suitable chemical modifications [6,11]. To improve the in vivo bioactivity of curcumin, a curcumin analogue (2*E*,6*E*)-2,6-*bis*-(4-hydroxy-3-methoxybenzylidene)-cyclohexanone (BHMC) was previously synthesized and characterized, and it has demonstrated significantly better in vivo anti-tumor and anti-metastasis effects than curcumin on murine ER negative 4T1 breast cancer cells [8]. A previous study has shown that cyclohexanone modification may further enhance the cytotoxicity of compounds on estrogen positive cancer cells [12]. However, to the best of our knowledge, the cytotoxicity effects of BHMC on the estrogen-positive breast cancer cells was not widely evaluated.

A previously discovered class of small (19–25 nucleotides) non-coding RNAs, known as the microRNAs (miRNAs) have been linked to the development of several human diseases [13]. Growing evidences have shown that several alterations in the miRNA profiles are involved in the progression of pathological conditions like cancer [14,15]. miRNAs represent an important group of non-coding RNA molecules due to their ability to regulate multiple downstream mRNA targets that play essential roles in regulating a vast range of cellular biological processes, including apoptosis [16,17]. Since miRNAs could act as crucial apoptosis regulators in tumorigenesis and cancer cells, these non-coding RNAs could also be potentially manipulated to regulate cancer cells survival and this strategy would help to improve sensitivity in cancer therapy [17].

In recent years, several published studies have reported that curcumin possesses the ability to regulate miRNAs expressions in cancers such as colon cancer [18] and lung cancer [19]. However, for curcumin analogue BHMC, not many studies have reported its potentials to regulate miRNAs expressions in cancers, particularly in human breast cancer. Therefore, this study was aimed to evaluate the ability of BHMC to regulate the miRNAs expression profiles in ER positive human breast cancer cell line, MCF-7. In addition, this study was also aimed to elucidate the potential of BHMC to induce apoptosis in MCF-7 and it was hypothesized that BHMC was able to regulate apoptotic signaling pathways in MCF-7 via a unique miRNA-mRNA interaction, which is yet to be reported elsewhere.

## 2. Results

### 2.1. BHMC Selectively Inhibits the Proliferation of MCF-7 Cell and MDA-MB231

To examine the selective cytotoxicity effect of BHMC on breast cells, MTT assay was conducted on both MCF-7 and MDA-MB-231 breast cancer cell lines as well as on the human normal breast MCF-10A cell line (Table 1). BHMC was shown to exhibit more superior cytotoxicity on both cancer cell lines, and the effect was found to be time dependent as the IC_50_ of BHMC on both cancer cell lines showed decreasing trend from 24 to 72 h. When comparing between MCF-7 and MDA-MB-231, it was observed that generally lower BHMC concentrations were needed to kill 50% of MCF-7 cells at three different timepoints (24, 48 and 72 h) as compared to MDA-MB-231. This suggested that BHMC might exhibit higher selectivity to kill ER positive MCF-7 than ER negative MDA-MB-231. When taking into consideration of selectivity index (SI) by dividing the IC_50_ of MCF-10A with IC_50_ of either cancer cell lines, it was shown that selectivity of BHMC was higher in MCF-7 (SI > 7) than MDA-MB-231 (SI > 5) against MCF-10A. At 24 h, the SI for MCF-7 was not statistically different (*p* > 0.05) to the SI for MDA-MB-231. However, as time progressed, the difference in the SI for MCF-7 and MDA-MB-231 became significant (*p* < 0.05) and this suggested that BHMC would induce a more selective cytotoxicity against MCF-7 than MDA-MB-231 at prolonged exposure. In short, the selectivity of BHMC on the three breast cell lines could be summarized as MCF-7 > MDA-MB-231 > MCF-10A.

On the other hand, when comparing BHMC and curcumin, it was observed that generally, BHMC exhibit better cytotoxic effects on the three human breast cell lines, evidenced by the recordings of lower IC_50_ of BHMC at three different timepoints on the three cell lines as compared to curcumin. However, curcumin shower higher SI for MDA-MB-231 than MCF-7. This implied that curcumin possessed more selective cytotoxicity against MDA-MB-231 than MCF-7. Besides, SI value of curcumin reduced across the time showing that selectivity of curcumin to both MCF7 and MDA-MB-231 against MCF-10A cells was reduced when the incubation time was prolonged.

### 2.2. Morphology Observation of MCF-7 Treated with BHMC

**Figure 1:**
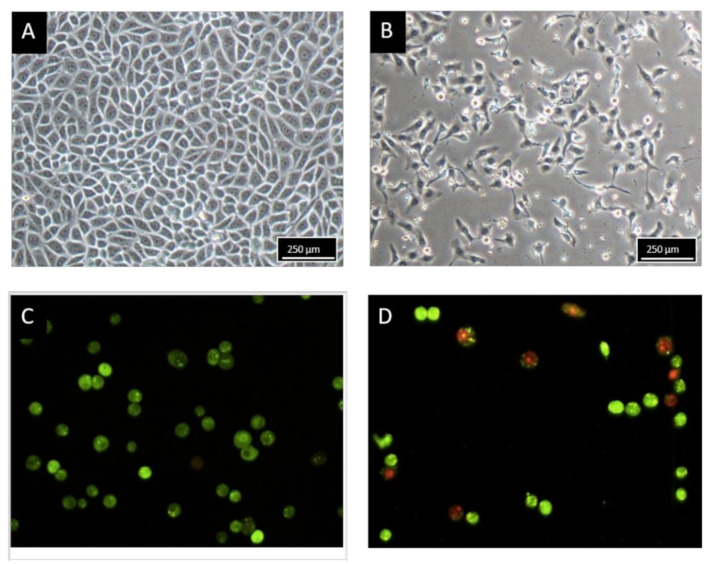
Morphological changes of MCF 7 cells viewed under light microscope after exposure to: (**A**) Untreated, (**B**) 8.2 µM of BHMC for 48 h. Fluorescent microscopy of acridine orange and propidium iodide dual staining of human breast cancer cells lines (MCF-7) (**C**) untreated and (**D**) 8.2 µM of BHMC for 48 h. Note: Figures shown were representative of one of at least three independent replicates with similar parameter (magnification 100×).

The morphological changes of untreated MCF-7 cells and MCF-7 cells treated with 8.2 µM of BHMC for 48 h are illustrated in Figure 1. With the 48 h of treatment, light microscopic observation (Figure 1B) revealed that BHMC reduced the MCF-7 cells number and severely distorted the MCF-7 cells shape. The treated MCF-7 cells underwent cell shrinkage and detachment. Meanwhile, the untreated MCF-7 cells (Figure 1A) displayed normal and polygonal cells shape evidenced by the clear and distinctive cell membrane boundary. Fluorescent microscopic examination was performed on MCF-7 cells stained with acridine orange and propidium iodide to assess the presence of apoptosis in both untreated and BHMC-treated MCF-7 cells. As shown in Figure 1C, untreated MCF-7 cells were viable and emitted green fluorescence light with clear, intact, and rounded shapes. In contrast, treated MCF-7 cells (Figure 1D) presented the morphological characteristics of apoptotic cells, which were evidenced by the presence of cells shrinkage and membrane blebbing as well as necrotic or late apoptotic morphological features, which emitted red fluorescence light.

### 2.3. BHMC Induced G2/M Cell Cycle Arrest Followed by Apoptosis on MCF-7

**Figure 2:**
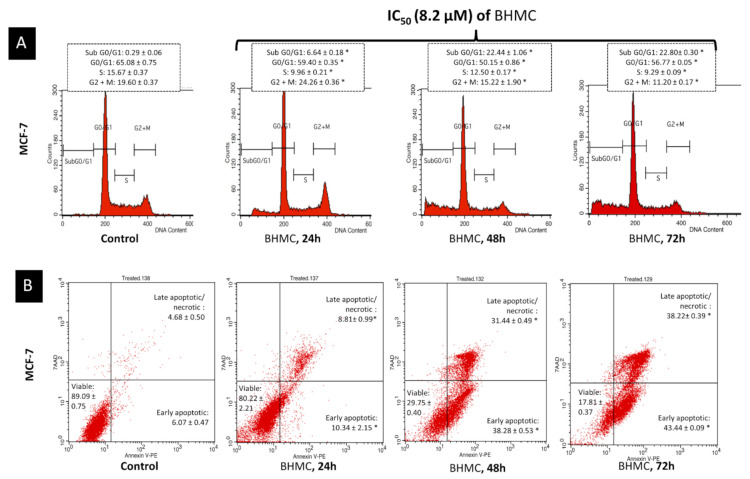
DK2 induces apoptosis in MCF 7 cells via G1/S cell cycle arrest. (**A**) Histogram analysis of the cell cycle machinery in MCF-7 after BHMC treatment for 24, 48, and 72 h. (**B**) Detection of phosphatidylserine (PS) exposure through the detection of Annexin V-PE and 7ADD fluorescence uptake in BHMC treated MCF-7 cells. Note: Values are mean ± SD of three replicates and significantly different from the untreated group (* *p* < 0.05) by ANOVA and followed by Duncan’s multiple range test. Figures shown are representative of one of at least three independent replicates with similar parameter.

To determine whether BHMC-induced growth inhibition was associated with the regulation of the cell cycle, the cell cycle distribution was analyzed using flow cytometry. As shown in Figure 2A, it was observed that there was an increase in the percentage of cells at G2/M phase from 19.60% to 24.26% after 24 h of BHMC treatment. This indicated the occurrence of cell cycle arrest with increment in the percentage of cells at subG_0_/G_1_ phase from 0.29% to 6.64% after 24 h of BHMC treatment. Accumulation of cells at the G2/M phase was accompanied by a significant increment in the percentage of hypodiploid cells of the subG_0_/G_1_ population from 6.64% at 24 h of treatment to 22.80% at 72 h of treatment. This possibly signified the occurrences of DNA fragmentation leading to cell death. To further confirm the presence of apoptosis-inducing cell death, BHMC-treated cells were stained with AnnexinV/7ADD and subjected to flow cytometry. Translocation of phosphatidylserine (PS) to the outer plasma membrane is a feature to recognize cellular apoptosis and this could be identified through the detection of Annexin V-7ADD fluorescence uptake in BHMC-treated MCF-7 cells. This method could be used to discriminate between early and late apoptosis. AnnexinV/7ADD staining (Figure 2B) revealed that from 24 to 72 h of BHMC treatment, there was an increase in the occurrences of early apoptosis from 10.34% to 43.44% and late apoptosis from 8.81% to 38.22%. This presumably suggested that BHMC treatment promoted G2/M arrest in early time point and subsequently induced apoptosis at a later time point.

### 2.4. BHMC Dysregulated miRNA and Gene Expression Profiles of MCF-7 Cells

**Figure 3:**
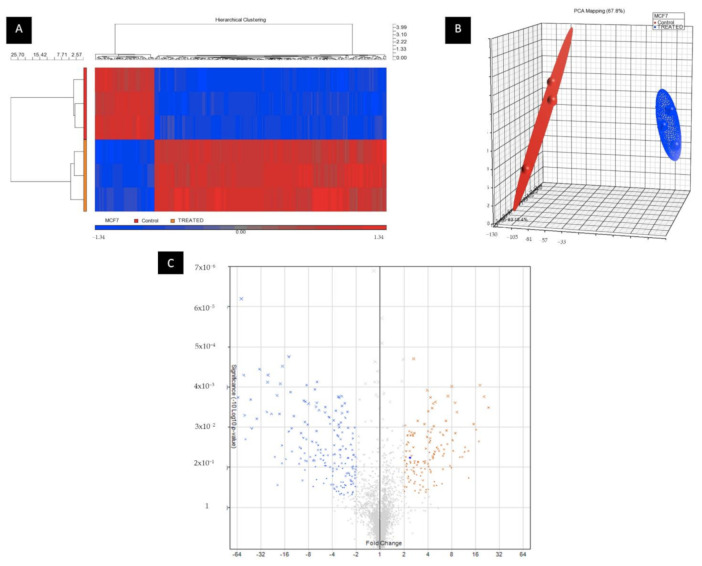
miRNA microarray data revealed differential gene expression between control and BHMC treated MCF-7 cell; (**A**) heatmap cluster analysis depicting differential miRNA (>2-fold change, *p* < 0.05) for BHMC treated cells and control MCF-7 cells. Up-regulated genes are depicted in red, down-regulated genes are in blue (see color bar); (**B**) principal component analysis plot; Tthe closer the dots, the more similar the gene expression profiles are; the farther apart the dots are, the greater the differences are; (**C**) volcano plot.

After normalization using Expression Console (Affymetrix, Santa Clara, CA, USA), differential analysis between control MCF-7 and BHMC-treated MCF-7 cells was performed using Transcriptome Analysis Console (TAC) 2.0 Software, (Affymetrix, Santa Clara, CA, USA) and Partek Genomics Suite software (Cat 4462922G, Partek Inc., St. Louis, MO, USA) (Figure 3). FDR multiple test correction was used for identifying differentially expressed genes between the two groups. Overall, 109 miRNAs were identified to be differentially expressed in the BHMC-treated MCF-7 cells under the threshold of *p* < 0.05 and fold-change > 5.

The top five miRNAs that were found to be overexpressed from miRNA microarray analysis include miR-184, miR-3195, miR-149-5p, miR-30a-3p, and miR532-3p and these miRNAs were found to be upregulated for at least 10-folds. Another five miRNAs were shown to be downregulated in the BHMC-treated MCF-7 cells and these include miR-6813-5p, miR-6132, miR-4725-3p, miR-1587, and miR-6779-5p. The expression of these miRNAs was shown to be downregulated for at least 30-folds.

### 2.5. Validation of Selected Genes and miRNAs by Quantitative Real-Time PCR (qPCR)

**Figure 4:**
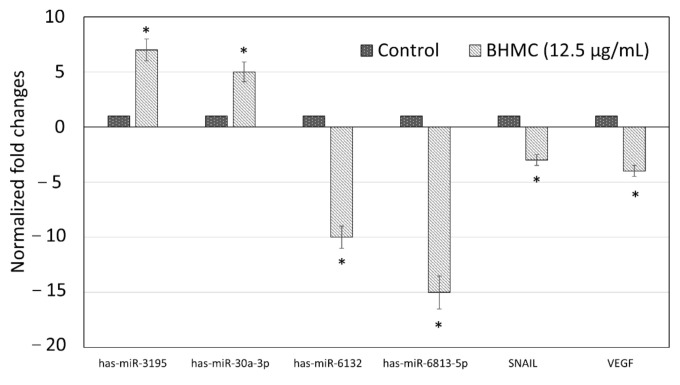
Validation of known miRNA and targeted genes using qPCR. Values are mean ± SD of three replicates and significantly different from the untreated group (* *p* < 0.05) by ANOVA and followed by Duncan’s multiple range test. Figures shown were representative of one of at least three independent replicates with similar parameter.

In order to validate the microarray data, qPCR analysis was conducted using 4 miRNAs and 2 target genes in which the expressions of these miRNAs and genes were altered by the BHMC treatment. The miRNAs that were selected for qPCR analysis include miR-3195 and miR-30a-3p (for upregulated miRNAs) and miR-6813-5p and miR-6132 (for downregulated miRNAs). Using miRSystem (http://mirsystem.cgm.ntu.edu), VEGF and SNAIL were the mRNA targets which were identified to be the downstream targets that could be regulated by miR-3195 and miR-30a-3p, and thus, these two targets were selected for further qPCR analysis. Compared to untreated MCF-7 cells (Figure 4), qPCR analysis revealed that both miR-3195 and miR-30a-3p were overexpressed in the BHMC-treated MCF-7 and the expression increments were at least 5-fold for both miRNAs. On the other side, qPCR analysis demonstrated that the expressions of both miR-6813-5p miR-6132 were downregulated for at least 10-folds in the BHMC-treated MCF-7 cells. As both miR-3195 and miR-30a-3p have been proven to be overexpressed in the BHMC-treated MCF-7 using microarray and qPCR data, it is therefore not surprising to observe that SNAIL and VEGF were also downregulated in the BHMC-treated MCF-7 cells using qPCR analysis, as these two targets were known to be the downstream targets of miR-3195 and miR-30a-3p. Overall, a high correlation between microarray (Table 2) and RT-qPCR data (Figure 4) was observed.

## 3. Discussion

BHMC has been reported to possess anti-tumor and anti-metastatic properties against ER negative 4T1 breast cancer cells in vivo [8], and anti-invasive properties against ER negative MDA-MB-231 breast cancer cells in vitro [20]. As the cytotoxic effects of BHMC on ER positive breast cancer cells were not widely evaluated and explored, this study was therefore aimed to determine the cytotoxic effects of BHMC on ER positive MCF-7 breast cancer cells. The cytotoxic effects of BHMC on MCF-7 were verified using both cell viability assay and light and fluorescence microscopic examinations. The current study discovered that BHMC exhibits selective cytotoxic effects on MCF-7 cells as compared to MDA-MB-231 cells and normal breast cancer cell line MCF-10A. Besides, the cytotoxicity assay also found that the selective cytotoxic effects of BHMC on MCF-7 cells were more evident when MCF-7 cells were exposed to BHMC for longer than 24 h. A previous report has highlighted that synthetic curcumin derivative was capable to induce selective cytotoxicity against human breast cancer cell than other cell types in a time-dependent manner [21]. Thus, it is hypothesized that different curcumin analogues would exert different selective cytotoxicity on different cell types when exposed at different duration length. On the other hand, as compared to BHMC, curcumin was found to exert more selective cytotoxicity against MDA-MB-231 than MCF-7. A previous report has demonstrated that a synthetic curcumin analogue with a structure that is related to BHMC called diferuloyl-(4-hydroxy-3-methoxycinnamoyl) moiety with mono-carbonyl was also found to exhibit potential cytotoxic effects against human breast cancer cells in vitro [22]. However, compared to BHMC, diferuloyl-(4-hydroxy-3-methoxycinnamoyl) moiety with mono-carbonyl was found to have better cytotoxic effects against MDA-MB-231 than MCF-7 cell lines [21]. This implied that diferuloyl-(4-hydroxy-3-methoxycinnamoyl) moiety with mono-carbonyl has similar selective cytotoxicity effects toward MDA-MB-231 than MCF-7 like curcumin, and synthetic curcumin analogue with different chemical structures and modifications might exhibit different selective cytotoxicity against different cells as described in the report [21].

To further prove that BHMC could promote cell growth inhibition on MCF-7 cells, cell cycle and apoptosis assays were conducted. The experiments found that BHMC was able to induce cell cycle arrest at G2/M phase at early time point, followed by the induction of apoptosis in MCF-7 cells at a later time point following BHMC administration. These effects were similar with other synthetic curcumin analogues including DK1 on breast cancer cells [22], A501 on lung cancer cells [23], and MC37 on colorectal cancer cells [24]. The ability of BHMC to induce cell cycle arrest and apoptosis supported the previous cell viability study finding, which suggested that BHMC is capable to produce cytotoxic effects on MCF-7 cells.

In addition, microarray and qPCR analysis showed that BHMC was involved in upregulating several miRNAs like miR-3195 and miR-30a-3p and downregulating miRNAs such as miR-6813-5p and miR-6132 in MCF-7 cells. Several synthetic curcumin analogues have been previously reported to involve in regulating miRNAs expressions in cancer cells [25,26]. Examples of these analogues include EF24 analogue, which was shown to target miR-21 in human melanoma and prostate cancer cells [26], and CDF analogue, which was found to increase expressions of miR-101, let-7, miR-26, and a few more miRNAs in pancreas cancer [25]. Curcumin, on the other hand, has been widely reported in regulating miRNAs expressions in different human cancers like breast, colorectal, lung, and oral cancers [27,28,29]. When focusing on the few miRNAs, which were dysregulated following BHMC treatment, miR-3195 has been found to possess anti-tumor activity in laryngeal cancer cell [30] and anti-angiogenic properties on prostate cancer cell [31]. Similarly, miR-30a-3p has been reported to possess anti-tumor properties in gastric and liver cancer cells [32,33]. Thus, both miR-3195 and miR-30a-3p could act as tumor-suppressing miRNAs, and the upregulation of these two miRNAs in MCF-7 cells following BHMC treatment was consistent with the cellular assay findings, which showed that BHMC exhibits cytotoxic effects on the cancer cells. As for the miRNAs that were downregulated following BHMC treatment, both miR-6813-5p and miR-6132 have been reported to promote insulin resistance in hepatocellular carcinoma cell line and, therefore, these miRNAs could be having tumor-promoting role [34]. miR-184, which was the most highly upregulated target in the BHMC-treated MCF-7 cells, was not selected for validation analysis as this miRNA was previously reported with both positive [35] and negative [36] regulation on the progression of different types of cancer cells. The microarray study also postulated that the role of miR-184 in breast cancer cells requires further investigation. Again, the downregulation of miR-6813-5p and miR-6132 in BHMC-treated MCF cells supported the findings, which showed that BHMC possesses cytotoxic effects on MCF-7 cells.

As VEGF and SNAIL have been identified to be the downstream targets of miR-3195 and miR-30a-3p (http://mirsystem.cgm.ntu.edu), qPCR analysis on these two genes were later performed and it was shown that the expressions of both VEGF and SNAIL were suppressed in the BHMC-treated MCF-7 cells. The VEGF signaling pathway in cancer cells is responsible in promoting carcinogenesis by promoting angiogenesis, invasion, migration, and apoptosis resistance [37] whereas SNAIL has been reported to promote breast cancer tumerigenesis by inducing epithelial-to-mesenchymal transition (EMT) [38]. Since both VEGF and SNAIL are tumor-promoting genes [37,38], the downregulation of these two targets by miR-3195 and miR-30a-3p secondary to BHMC treatment would probably induce cellular death in the MCF-7 cells. Subsequent pathway analysis showed that several cellular biological pathways like p53 and Wnt signaling pathways were dysregulated in the BHMC-treated MCF-7 cells. p53 is a tumor-suppressing protein that plays an essential role in inducing apoptosis [39] while Wnt signaling pathway has been demonstrated to regulate early and late phases of apoptosis in human cells [40]. Dysregulation of these apoptosis-related pathways possibly triggered the occurrence of cellular suicide in the BHMC-treated MCF-7 cells.

## 4. Materials and Methods

### 4.1. Source of Curcumin and Preparation of BHMC

**Figure 5:**
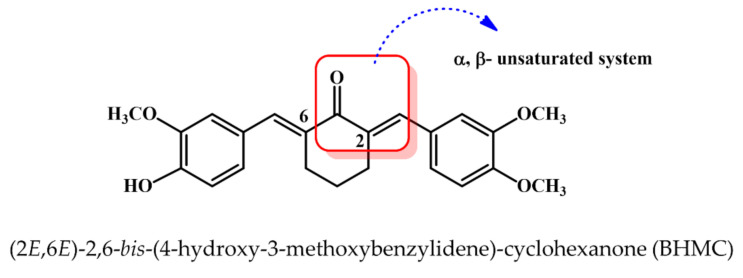
Chemical structure of BHMC [8].

Curcumin used in this study was of analytical grade and was purchased from Sigma-Aldrich (St. Louis, MO, USA). BHMC (Figure 5) was synthesized and characterized as previously reported [8].

### 4.2. Breast Cells Culture Conditions

Estrogen-positive MCF-7 and estrogen-negative MDA-MB-231 breast cancer cells were maintained in RPMI 1640 (Sigma, St. Louis, MO, USA) or DMEM (Sigma, USA), respectively, supplemented with 10% fetal bovine serum (FBS) (Gibco Thermo Fisher Scientific, Waltham, MA, USA). Normal MCF-10A cell was maintained in DMEM-F12 (Sigma, USA) supplemented with 0.5 μg/mL hydrocortisone, 10 μg/mL insulin, 20 ng/mL human epidermal growth factor (hEGF) (Sigma, USA), and 10% FBS (Gibco Thermo Fisher Scientific, Waltham, MA, USA). All cells were cultured at 37 °C in 5% CO_2_ environment and the cells passage ranged from passage 10 to 20. In addition, all breast cells used were negative for mycoplasma growth. Upon reaching 80% confluency, the corresponding adherent cells were harvested using TypLE (Thermo Fisher Scientific, Waltham, MA, USA) for subsequent experiments.

### 4.3. MTT Cell Viability Assay

The MTT cell viability assay for MCF-7, MDA-MB-231 and MCF 10A cells were conducted according to a previously reported protocol [41]. MCF-7, MDA-MB-231, and MCF-10A were seeded in a 96-well plate at a concentration of 0.8 × 10^5^ cells/mL and left overnight in a CO_2_ incubator set at 37 °C. Then, BHMC with 2-fold dilution ranging between 180 and 3 µM was added to the respective cell lines while the last row of the 96-well plate served as untreated control cells. After 24, 48, and 72 h of incubation, 20 µL of 5 mg/mL MTT solution was added to all wells and the plate was further incubated for three hours. Then, 180 µL of supernatant was carefully discarded and added with 100 µL of DMSO to solubilize the purple crystals. Optical density was measured at 570 nm wavelength using ELISA Reader (Bio-tek Instrument, Winooski, Vermont, USA). The percentage of cell viability was calculated following deduction of the blank cell absorbance using the formula (Equation (1)) [42]:

(1) $$\mathrm{Cell}\;\mathrm{viability}\;\left(\%\right)\;=\frac{\mathrm{Absorbance}\;\mathrm{of}\;\mathrm{treated}\;\mathrm{cells}}{\mathrm{Absorbance}\;\mathrm{of}\;\mathrm{untreated}\;\mathrm{control}}\times \;100\%$$

The dose–response curve was plotted, and the concentration that yields 50% inhibition of cell growth (IC_50_) was obtained as a parameter for cytotoxicity. Starting from this point, BHMC with IC_50_ value was used throughout the study to induce cell death. On the other hand, selectivity index (SI) was obtained by using the following formula (Equation (2)) [43]:

(2) $$\mathrm{Selectivity}\;\mathrm{index}\;\left(\mathrm{SI}\right)\;=\frac{{\mathrm{IC}}_{50}\;\mathrm{of}\;\mathrm{MCF}-10A}{{\mathrm{IC}}_{50}\;\mathrm{of}\;\mathrm{MCF}-7\;\mathrm{or}\;\mathrm{MDA}-\mathrm{MB}-231}$$

### 4.4. MCF-7 Cell Treatment

MCF-7 cells were seeded at 0.8 × 10^5^ cells/mL in a 6-well plate and left overnight in a CO_2_ incubator set at 37 °C. After reaching 80–90% confluent, the cell was treated with IC_50_ at 48 h (12.50 µM) of BHMC. Untreated control was prepared simultaneously. After 24, 48, or 72 h of incubation, the cell was harvested using TrypLE (Gibco Thermo Fisher Scientific, Waltham, MA, USA), washed with phosphate buffer saline (PBS), and subjected to the following assays. All assays were tested with three biological replicates.

### 4.5. Light and Fluorescent Microscopic Observation

Morphology of control and BHMC-treated MCF-7 cells was observed under light microscope (Nikon, Minato City, Japan). For fluorescent microscope observation, control and BHMC-treated MCF-7 cells (2 × 10^5^ cells) were stained with 10 μL of 100 μg/mL acridine orange (AO) and 100 μg/mL propidium iodide (PI) for 10 min. After that, the cells were washed and examined under fluorescent microscope (Nikon FC-35DX, Minato City, Japan) using an excitation filter and barrier filter at 450–490 nm and long pass filter of 520 nm.

### 4.6. Flow Cytometry Cell Cycle Analysis

Cell cycle progression of control and BHMC-treated MCF-7 cells was evaluated by staining with BD Cycletest Plus kit (Becton Dickinson, Franklin Lakes, NJ, USA) and subjected to BD FACS Calibur flow cytometer (Becton Dickinson, USA) analysis. In brief, harvested cells were incubated with 250 µL of trypsin buffer for 10 min followed by addition of 200 µL of trypsin inhibitor containing RNase buffer. After 10 min of incubation, the samples were finally stained with 200 µL of PI solution. Flow cytometer analysis was carried out after 10 min of incubation with the PI stain solution. A minimum of 10,000 cells in the population was captured. Three independent experiments were repeated with similar parameter.

### 4.7. Flow Cytometry AnnexinV/PI Apoptosis Detection

Cell apoptosis assay was conducted based on a study method [44] with some changes. Apoptosis of control and BHMC-treated MCF-7 cell was evaluated using AnnexinV-FITC/PI apoptosis detection kit (Becton Dickinson, USA). In brief, harvested cells were washed and re-suspended in 100 µL of PBS. Then, the cell was stained with 10 µL of AnnexinV-FITC/PI solution and incubated for 20 min in dark prior to analysis with BD FACS Calibur flow cytometer (Becton Dickinson, USA). Approximately, 10,000 cells in the population were captured. The experiment was qualitatively repeated for three times.

### 4.8. Total RNA and miRNA Extraction

Total RNAs including small RNAs were isolated from MCF-7 treated with BHMC using miRNeasy kit (Qiagen, Hilden, Germany) according to manufacturer’s instructions. The quality of extracted RNA was assessed by the NanoDrop-1000 Spectrophotometer (NanoDrop Technologies Inc., Wilmington, DE, USA) and Agilent 2100 Bioanalyzer, and samples with A260/A280 ratio between 1.8 to 2.1 and an RIN value of 8 and above was used for further analysis.

### 4.9. Microarray Analysis of miRNA

Total RNA (8 µL) of control and BHMC-treated MCF-7 cells was directly labelled using Flash Tag Biotin HSR Labeling kits (Affymetrix, Santa Clara, CA, USA) in accordance with the instructions of the manufacturer. RNA was heated to 80 °C for 10 min before labeling to inactivate any residual DNase activity. RNA was hybridized for 42 h to the GeneChip miRNA 2.0 array (Affymetrix, Santa Clara, CA, USA). The GeneChip miRNA 2.0 arrays contain 100% miRBase version 15 coverage of 131 organisms and contain probes for 3439 humans non-coding RNAs (ncRNAs), including 1105 miRNAs and 2334 other ncRNAs (including scaRNAs and snoRNAs). Washing and staining were automatically performed using the Affymetrix Fluidics Station 450 (Affymetrix, Santa Clara, CA, USA), and probe intensities were measured using the GeneChip^®^ Scanner (Affymetrix, Santa Clara, CA, USA).

### 4.10. Analysis of Microarrays Data

Data discussed in this publication have been deposited in the NCBI Gene Expression Omnibus and are accessible through the GEO Series accession number GSE155467. Using the gene expression workflow in Partek Genomics Suite software (Cat 4462922G, Partek Inc., St. Louis, MO, USA), the CEL files generated by the Affymetrix data file were converted into .nFMT files. These data were later converted to .XLS files. and normalized using the robust multi-array average (RMA) normalization procedure as described in a published study [45]. Differentially expressed miRNAs (>5-fold change, *p* < 0.05) were identified using analysis of variance (ANOVA) analysis. A list of differentially expressed miRNAs between the control and BHMC-treated MCF-7 cell was exported in the table form to an excel file and was also displayed in the form of heat map to visualize the list of up- and down-regulated miRNAs between the two groups.

### 4.11. Real-Time Quantitative PCR of miRNAs Expression

One microgram (μg) of total RNA was used in reverse transcription and the process was conducted using the miScript II RT Kit (Qiagen/SABiosciences, Hilden, Germany) according to the manufacturer protocol. Quantitative RT-PCR was carried out using CFX96 (BioRad, Hercules, CA, USA). RNU-6 (Qiagen, USA) was used as the internal control as reported in the other study [46]. miScript SYBR Green PCR kit (Qiagen/SABiosciences, Hilden, Germany) was used in the real-time PCR reaction according to the manufacturer’s suggested steps. The miRNA-specific primers for miR-3195, miR-30a, miR-6813-5p, and miR-6132 were designed by miRprimer and commercially synthesized as listed in Table 3. The expression of putative miRNAs was evaluated using geNorm algorithms. PCR reactions were performed in triplicate for each sample. The relative amounts of miRNAs were normalized against reference miRNAs and the fold change for each miRNA was calculated by the 2^−ΔΔCt^ method [47].

### 4.12. Real-Time Quantitative PCR of mRNA Expression

Real-time quantitative PCR was performed on SNAIL and VEGF, which were the target of miR-30a [48] and miR-3195 [31]. One microgram (μg) of total RNA was reverse transcripted using the QuantiTect Reverse Transcription Kit (Qiagen, USA) according to the manufacturer protocol. Primers were designed using Primer-Blast (Table 2) and synthesized commercially. NEXpro qPCR Evagreen Master Mix (NEX Diagnostics, Gyeonggi-do, Korea) was used to perform the mRNA qPCR based on the manufacturer protocol. The qPCR system CFX96 (BioRad, USA) was used and the qPCR conditions were: 50 °C for 2 min, 95 °C for 10 min, and 40 cycles of 95 °C for 15 s, and 60 °C for 1 min. A final melting curve was performed to ensure that only one amplicon was present. The relative amounts of mRNAs were normalized against reference gene beta-actin (ACTB) as described previously [49] and the fold change for each mRNA was calculated by the 2^−ΔΔCt^ method [47]. The mRNA primers sequences were listed in Table 4.

### 4.13. Statistical Analysis

Data are expressed as means ± standard deviations (SD). Statistical analyses were performed using one-way analysis of variance (ANOVA) and were compared by FDR correction and Duncan’s post hoc test. The results were taken to be significant at a probability level of *p* < 0.05.

## 5. Conclusions

This study showed that BHMC is capable to induce cytotoxic effects on the ER positive human breast cancer cells, MCF-7, and alter the expressions of multiple miRNAs and genes in the cancer cells. The dysregulation of these miRNAs and the associated downstream targets were believed to trigger the occurrence of apoptosis in the MCF-7 cells. To validate the in vitro findings reported in this study, we hope that in vivo and mechanistic study could be performed in the near future to further prove that BHMC is able to initiate breast cancer cells apoptosis by altering the expressions of apoptotic-regulating miRNAs and associated genes.

## Figures and Tables

**Table 1:** IC_50_ values and selectivity index (SI) of MCF-7, MDA-MB-231, and MCF-10A treated with BHMC or curcumin. IC_50_ was reported as mean + standard deviation (SD) and the results were generated from three biological replicates, and each biological replicate contained three technical replicates. SI: Selectivity index.

Cell Lines	Treatment	24 h	48 h	72 h
MCF-7	BHMC (µM)	23.50 ± 2.41	12.50 ± 1.87	10.98 ± 1.33
	Curcumin (µM)	55.72 ± 2.77	45.15 ± 2.12	39.79 ± 1.51
MDA-MB-231	BHMC (µM)	29.35 ± 3.15	20.05 ± 1.94	19.69 ± 3.14
	Curcumin (µM)	32.00 ± 2.81	26.00 ± 2.33	23.00 ± 2.15
MCF-10A	BHMC (µM)	180.00 ± 3.11	108.00 ± 2.15	98.40 ± 3.22
	Curcumin (µM)	188.00 ± 4.52	112.00 ± 4.57	100.00 ± 4.31
SI MCF10A/MCF7	BHMC (µM)	7.66	8.64	8.96
	Curcumin (µM)	3.37	2.48	2.51
SI MCF10A/MDA-MB-231	BHMC (µM)	6.14	5.38	5.00
	Curcumin (µM)	5.88	4.31	4.35

**Table 2:** Top five up- and down-regulated miRNAs significantly altered in BHMC-treated MCF7 cell.

Transcript ID	Upregulated Fold Change	Transcript ID	Downregulated Fold Change
hsa-miR-184	17.58	hsa-miR-6779-5p	−33.07
hsa-miR-3195	16.21	hsa-miR-1587	−35.89
hsa-miR-149-5p	13.06	hsa-miR-4725-3p	−41.51
hsa-miR-30a-3p	12.9	hsa-miR-6132	−61.96
hsa-miR-532-3p	11.73	hsa-miR-6813-5p	−71.82

**Table 3:** List of miRNA-specific primers used in the study.

MicroRNA Targets	Primer Sequence (5′–3′)
has-miR-3195	F: GCGCCGGGCCC
	R: CAGGTCCAGTTTTTTTTTTTTTTTAAC
has-miR-30a-3p	F: GCTTTCAGTCGGATGTTTG
	R: GGTCCAGTTTTTTTTTTTTTTTGCT
has-miR-6132	F: GCAGGGCTGGGAT
	R: GGTCCAGTTTTTTTTTTTTTTTGCA
has-miR-6813-5p	F: AGGGGCTGGGGTTTC
	R: CCAGTTTTTTTTTTTTTTTAGAACCTG

**Table 4:** Primer sequence of the gene detected in the qPCR assay.

Gene	Primer Sequence (5′–3′)
*SNAIL*	F: GCCGACTTTTGTGGTCTTCC
	R: GGTACAAGTATGCCTCTGCCA
*VEGF*	F: GCTGTGGACTTGAGTTGGG
	R: GCTGGGTTTGTCGGTGTT
*ACTB*	F: AGAGCTACGAGCTGCCTGAC
	R: AGCACTGTGTTGGCGTACAG

## Data Availability

Data discussed in this publication have been deposited in the NCBI Gene Expression Omnibus and are accessible through the GEO Series accession number GSE155467.

## Sample Availability

Samples of the compound are available from the authors upon discussion.
